# Immune responses to malaria pre-erythrocytic stages: Implications for vaccine development

**DOI:** 10.1111/pim.12795

**Published:** 2020-10-09

**Authors:** Kelvin Mokaya Abuga, William Jones-Warner, Julius Clemence R. Hafalla

**Affiliations:** 1 Department of Infection Biology, Faculty of Infectious Diseases, London School of Hygiene and Tropical Medicine, London, UK; 2 Department of Epidemiology and Demography, KEMRI-Wellcome Trust Research Programme, Kilifi, Kenya

**Keywords:** exoerythrocytic forms, liver, malaria immunology, *Plasmodium*, pre-erythrocytic, RTS,S, skin, sporozoite

## Abstract

Radiation-attenuated sporozoites induce sterilizing immunity and remain the ‘gold standard’ for malaria vaccine development. Despite practical challenges in translating these whole sporozoite vaccines to large-scale intervention programmes, they have provided an excellent platform to dissect the immune responses to malaria pre-erythrocytic (PE) stages, comprising both sporozoites and exoerythrocytic forms. Investigations in rodent models have provided insights that led to the clinical translation of various vaccine candidates—including RTS,S/AS01, the most advanced candidate currently in a trial implementation programme in three African countries. With advances in immunology, transcriptomics and proteomics, and application of lessons from past failures, an effective, long-lasting and wide-scale malaria PE vaccine remains feasible. This review underscores the progress in PE vaccine development, focusing on our understanding of host-parasite immunological crosstalk in the tissue environments of the skin and the liver. We highlight possible gaps in the current knowledge of PE immunity that can impact future malaria vaccine development efforts.

## 1 Background

Malaria remains an intractable global public health problem with an estimated 228 million cases and 405 000 deaths in 2018 alone.^1^ A vast majority of these deaths occur in sub-Saharan Africa, where malaria is associated with a 24% prevalence and 94% of the malaria-associated deaths globally.^1,2^ Recent advances in malaria control including improved diagnostic approaches, artemisinin-combination treatments (ACTs), intermittent preventive treatment (IPT) in pregnancy and vector control saw a 48% decrease in mortality rates between 2000 and 2015.^1^ Whilst these strategies have unquestionably contributed to reduction in incidence and mortality rates, an effective vaccine would provide the ultimate solution to malaria elimination and should be an urgent public health priority.

**Figure 1:**
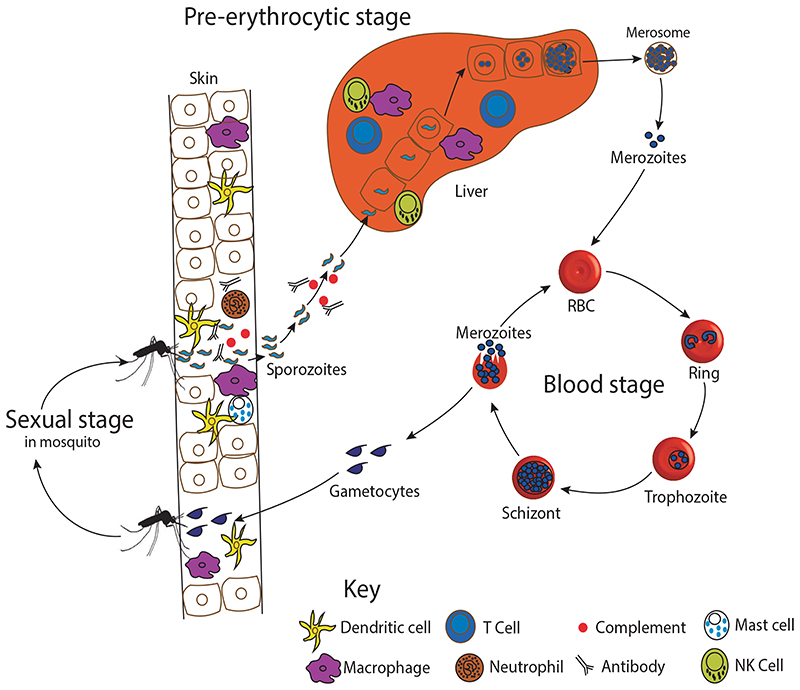
The malaria life cycle. An infected mosquito deposits motile infective sporozoites into the dermis of a susceptible host. Some sporozoites migrate to the liver, where they invade hepatocytes, multiply asexually to produce thousands of merozoites which egress in merosomes and rupture inside microvasculature of lungs. The merozoites invade the red blood cells (RBC), and undergo multiple cycles of ring, trophozoite and schizont stages, to initiate the clinical phase of the disease. Some parasites differentiate into male and female gametocytes, which are taken up mosquitoes during their next blood meal. Different immune cells interact with the malaria sporozoites during its journey from the skin to the liver and may be exploited in the development of an effective and long-lasting vaccine. NK denotes natural killer cells

Malaria biology is complex. Our understanding of the pre-erythrocytic (PE) stage infections is based on model systems with *Plasmodium berghei* (*Pb*) and *P. yoelii* (*Py*), with limited information on *P. falciparum* (*Pf*). The PE stage begins when an infected female *Anopheles* mosquito inoculates a few (typically <100) infective sporozoites into the host skin.^3,4^ Quantitative studies with *Pb* and *Py* indicate that a large proportion (~60%) lose motility and remain localized at the site of inoculation where they can develop into skin exoerythrocytic forms (EEF) and initiate an immune response.^5–8^ Some sporozoites ‘trickle out’ of the skin into the blood (~25%) and the lymphatic drainage (~15%).^6,9^ Most of the sporozoites that enter the bloodstream reach and invade the liver, where they traverse through several hepatocytes in a transient vacuole. The sporozoites then invade a final hepatocyte and form parasitophorous vacuoles (PV), where the liver EEFs develop.^6,10^ The circumsporozoite protein (CSP), which is the major antigen on the sporozoite surface, and thrombospondin-related anonymous protein (TRAP), a micronemal protein, are thought to facilitate invasion into the hepatocytes.^11,12^ In the liver, the parasites undergo asexual development for a number of days depending on the *Plasmodium* species (ie 7-10 days for human malaria vs 42-44 hours for *Pb* infection in mice), pre-existing immunity and concomitant malaria prophylaxis.^13^ They differentiate into multinucleated schizonts that form thousands of merozoites via nuclear division. In the late stages of development, the PV membrane is lysed, and the merozoites become packaged together inside merosomes.^14,15^ These merosomes egress out of the liver, circulate through the heart and reach the lung microvasculature where merozoites are released to invade erythrocytes.^16^ This initiates bloodstage cycle of development amongst ring, trophozoite and schizont forms (Figure 1). Exponential expansion of the parasite during the blood-stage stage and concomitant immune responses result in malaria-related symptoms (as reviewed elsewhere).^17^

The PE stages form a bottleneck for the malaria parasite and can be targeted in developing an effective malaria vaccine. Once thought to be immunologically quiescent, accumulating evidence shows that the PE stages provoke immune responses.^8,18–21^ The sporozoites are exposed to antibodies in the bloodstream and in the skin and hepatic extracellular fluids. It is only during the PE stages where *Plasmodium* parasites invade nucleated cells of humans and rodent models, which can present parasite antigens via major histocompatibility complex (MHC) I. This gives a wide array of innate and adaptive immune effector mechanisms that can be exploited in developing an effective malaria vaccine. A vaccine targeting the clinically ‘silent’ PE stages will not only block symptomatic blood-stage infections and associated complications, but it would also halt further transmission of the parasite. Nonetheless, the host-parasite crosstalk during the PE stages is intricate and remains inadequately studied. In this review, we systematically explore the current knowledge on vaccine development and immune responses to malaria PE stages, and highlight some of the existing gaps.

## 2 Progress With Malaria Pre-Erythrocytic Stage Vaccines

### 2.1 Whole sporozoite vaccines

Seminal studies in the late 1960s on mice immunized with radiation-attenuated sporozoites (RAS) demonstrated sterile immune protection against malaria reinfections.^22,23^ The sterile protection was later observed in nonhuman primates and challenge human malaria infection (CHMI) trials with efficacy levels of >80%.^24–27^ RAS are currently in clinical trials across the world (Table 1). Due to the development of sterile immunity, RAS became the ‘gold standard’ for a malaria vaccine development. Nonetheless, translation of RAS to a wide-scale applicable human vaccine remains challenging. Extremely large numbers of dissected parasites (up to 6.75 × 10^5^ given at five doses or 9 × 10^5^ given at three doses), which are delivered intravenously, are required to induce sterile immunity.^28,29^ Increasing the dose to 1.8 × 10^6^ parasites greatly reduces vaccine efficacy. The sterile immunity induced by RAS is not long-lasting, but the durability of protection can be extended by booster immunizations.^26^

During the past two decades, there has been a renaissance of approaches to develop whole sporozoite vaccines. Pre-clinical evidence suggests that invasion and development in the liver are required for sterile PE immunity.^30,31^ RAS vaccines successfully invade the liver, but their development is arrested early in EEF development. Administration of sporozoites followed by antimalarial chemoprophylaxis with chloroquine or mefloquine (CPS vaccines), which acts on blood stage but not liver-stage parasites, yields comparable efficacy levels to RAS and confers protection against PE stages in both rodent models and humans.^32–36^ CPS vaccines may provide more robust immunity as the sporozoites undergo complete liver-stage development. Alternative CPS approaches involve using antibiotics, such as clindamycin and azithromycin, which allow full parasite development in the liver, but lead to delayed death in the resulting merozoites.^37^ In rodent models, CPS vaccines have been shown to induce robust, long-lived immunity that not only protects against PE stages, but also against blood stages.^38,39^ This apparent cross-stage immunity induced by CPS vaccines needs to be further explored.

Genetically attenuated parasite (GAP) vaccines rely on targeted gene deletion technology that arrests the development of sporozoites at either early- or late-stage EEFs. Studies initially targeted the attenuation of the upregulated in infective sporozoite (*uis*) genes, which attenuates sporozoite development in the early stages.^40,41^ *Pb* parasite lines with *uis3* ^-^ and *uis4* ^-^ knockout genes arrest their development after completion of sporozoite development in the early EEF stages.^42^ Studies using *Pb* found a stage-specific durable sterile protection against reinfection after immunization with three doses of *uis3^–^* sporozoites.^41^ GAP vaccines targetting other *Plasmodium* genes have produced varied results (as reviewed by Kreutzfeld et al^43^). A clinical trial using *Pf*GAP lacking two genes (*p36* ^-^ *p52* ^-^) reported favourable anti-sporozoite immune responses.^44^ The triple gene knockout (*Pf*GAP3KO: *p36* ^-^ *p52* ^-^ *sap1* ^-^) *Pf*GAP was reported to fully attenuate sporozoite development in the early liver stages in in vitro and humanized mice studies.^45,46^ *Pf*GAP3KO was reported to be safe and immunogenic in human volunteers after 150-200 mosquito bites, but is yet to complete clinical trials.^47^ Other GAP vaccine development efforts include targeting the late EEF stages, such as deletion of *fabb/f*, *PlasMei*2, and liver-specific protein 2 (*LISP2*) genes.^31,48^ GAP vaccines targeting the late EEF stages may be efficacious at lower doses, induce a larger breadth of immune responses and protect against blood-stage infections.^49^ *Pf*SPZ-GA1 vaccine, a *Pf* identical double knockout (*b9* ^-^ *slarp* ^-^), which attenuate early in EEF development, presented safety profile and elicited immune responses.^50^ The pre-clinical findings of PfSPZ-GA1 are promising, as they have shown optimal immunogenicity and some indication of protection.

Sterile and cryopreserved sporozoite vaccines (*Pf*SPZ), injected intravenously, conferred up to 100% sterile protection after CHMI with homologous strains, and ~80% protection against heterologous strains.^51–54^ A comparable outcome is obtained with CPS vaccines (using chloroquine as the antimalarial drug) where only modest protection was obtained with heterologous challenge.^55^ A challenge for whole sporozoite vaccines is to increase the diversity of strains represented in the vaccine. Of particular interest, the inoculation of *Pf*SPZ intradermally, mimicking the natural route of sporozoite infection, was not protective.^54^ Additionally, *Pf*SPZ efficacy was greatly reduced in a setting of seasonal transmission, showing about 30% protection at 6 months in Mali adults.^56^ The low efficacy has been associated with hypo-responsiveness to *Pf*SPZ in malaria-exposed adults. A study on adult males from Equatorial Guinea reported lower antibody responses to *Pf*SPZ compared to US adults receiving a similar dosage regimen.^57^ Additional studies are required on dosage optimization for participants in malaria-endemic areas,^29^ and particularly for children who are most affected by severe malarial disease in sub-Saharan Africa. The need for liquid nitrogen storage to maintain *Pf*SPZ vaccines may be a logistical challenge in malaria-endemic areas. Future efforts should focus on developing a thermal-stable *Pf*SPZ vaccine, which can reduce delivery challenges to remote areas.

### 2.2 Sub-unitvaccines

Sporozoites are covered with a dense coat, and CSP—a 40-66 kDa protein, with ~40 NANP repeats in the central region of *Pf*CSP — is the major surface protein.^58^ Inadvertently, many approaches have been explored to target and improve immune responses to CSP. RTS,S/AS01 (Mosquirix™), the most advanced malaria vaccine to date, contains a section of the CSP central repeat region (18 NANP repeats with B-cell epitopes) and C-terminal (with T-cell epitopes). In a large phase III study involving 15 459 infants (6-12 weeks old) and young children (5-17 months old) at 11 sites, RTS,S showed moderate vaccine-induced protection at 18 months (26% and 45%, respectively) which waned on follow-up.^59^ In subjects receiving a booster at 20 months, the vaccine efficacy was ~36% in children (vs 28% in controls without the booster) and ~25% in infants (vs 18% in controls) at the end of a 48-month study period.^60^ Fractional dosing of the third dose may increase the vaccine efficacy up to ~86%,^61^ but this remains to be seen in endemic areas where efficacy in adult declined with an increase in malaria transmission.^62^ After a positive review by the European Medicines Agency, RTS,S was recently rolled out for implementation in three African countries (Malawi, Kenya and Ghana).^63^ Assuring earlier concerns that CSP diversity may impact vaccine efficiency, it is noteworthy that in the above large phase III trials, <10% of the parasites corresponded the CSP alleles used in the RTS,S.^64^

Prime-boost viral vectored delivery platforms using chimpanzee adenoviruses (eg ChAd63) prime and a modified vaccinia strain Ankara (MVA) have been explored as alternative approaches to improve the efficacy of CSP-based vaccines. ChAd63-MVA CSP vaccine candidate induced high levels of antigen-specific antibodies and T-cell responses.^65^ Nevertheless, its efficacy in a CHMI trial was poor, protecting only 1/15 subjects.^66^ In vitro and rodent studies have suggested that CSP is dispensable in achieving sterile immunity and low levels of anti-CSP antibodies may aid in sporozoite invasion.^58,67,68^ Other studies reported that the CSP repeat region, but not the C-terminal domain, induced antibody-dependent phagocytic activity that was protective against infection.^69^ Thus, the modest protection induced by CSP-based vaccines, as compared to the sterile immunity observed in RAS, calls for exploration of alternative adjuvants, antigens and/or CSP epitopes as vaccine targets and increased focus on antibody functionality rather than quantity.

The genome of *Pf* reference strain 3D7 contains ~5400 genes.^70^ Some of these genes encode for proteins that are essential for cell traversal (sporozoite microneme protein essential for cell traversal [SPECT], phospholipase [PL], cell-traversal protein for ookinetes and sporozoites [CelTOS], gamete egress and sporozoite traversal protein [GEST] and perforin-like protein [PLP1 also known as SPECT2]); liver invasion (TRAP and apical membrane antigen [AMA] 1) and hepatic development (liver surface antigens [LSA1, LSA2 and LSA3] and sporozoite threonine and asparagine-rich protein [STRAP]). Most of these proteins have the potential of becoming vaccine targets, but only a few are in current clinical trials (Table 1). ChAd63-MVA ME-TRAP, which primarily targets TRAP but also contains multiple epitopes of CSP, LSA1, LSA3, STARP, EXP1, has been reported to have high immunogenicity and safety levels in human studies even when administered concurrently with the expanded program on immunization.^71–74^ Combination vaccines of ME-TRAP and CSP have so far yielded varying results depending on vaccine regimen and routes of administration.^75–77^

## 3 Immune Responses To Malaria Pre-Erythrocytic Stages In The Skin And The Liver

### 3.1 Innate host responses in the skin and the liver

The skin is the first defence layer against the malaria parasites. Apart from being a physical barrier, the skin harbours a diverse range of phenotypically and functionally distinct dendritic cells (DCs) and macrophages that interact with sporozoites, as described in mouse malarias (Figure 1).^5,6^ The contribution of these cells is challenging to study in humans considering the ‘silent’ clinical nature of malaria PE stages. Neutrophils and monocytes infiltrate the site of sporozoite inoculation, and mast cells have been reported to induce DCs and T-cell recruitment.^78,79^ Remarkably, a rodent study reported that neutrophils and monocytes may not be critical in the development of sterile immunity.^78^ Further work is needed to dissect the roles of neutrophils and monocytes in PE stage immunity.

Whilst the liver is known to be an autonomous and competent priming site for naïve CD8^+^ T cells,^80^ the role of hepatocytes and other liver cells in antigen presentation during PE stages remain poorly understood. Liver cells including hepatocytes, liver sinusoidal endothelial cells, Kupffer cells, hepatic DCs and hepatic stellate cells interact with the parasite during the liver invasion process (as reviewed by Hafalla et al^81^). Rodent studies have shown that CD11c^+^ DCs found in the spleen, liver and liver-draining lymph nodes are required to present antigens to CD8^+^ T cells, and their depletion abrogates CD8^+^ T-cell responses.^5,82–84^ It is thought that these DCs directly present sporozoite antigens to CD8^+^ T cells through antigen cross-presentation.^5,8,83^ Blocking the ability of the DCs to cross-present antigens represses CD8^+^ T-cell responses.^85,86^ CD4^+^ T cells play a role in ‘licensing’ these antigen-presenting DCs.^83,87^ How antigens that are expressed exclusively during EEF development prime CD8^+^ T-cell responses remains inadequately characterized. Recent studies have implicated a subset of liver-infiltrating monocyte-derived CD11c^+^ cells, which acquire rodent parasites after parasite invasion but before merozoite release.^82^ Consistent with the presentation of sporozoite-derived antigens, these monocyte-derived CD11c^+^ cells were found to prime CD8^+^ T-cell responses in the liver-draining lymph nodes.

Infected hepatocytes can become ‘stressed’ (express heat shock proteins) and/or apoptotic.^21^ This induces inflammatory responses and recruitment of effector immune cells to the site of EEF infection. Plasmodial dsRNA accessing hepatocytic cytosol induces release of type I interferons (IFN-α and IFN-β) that recruit natural killer (NK) and CD3^+^CD49b^+^ natural killer T (NKT) cells.^88,89^ NK cells are a highly enriched effector cell population that respond to invading sporozoites, as they account for up to 50% of liver-resident lympho-cytes.^90^ NK and NKT cells are potent producers of IFN-γ,^18,20^ which activates the nitric oxide pathway in macrophages.^18,91^ In RTS,S CHMI studies, concentrations of serum IFN-γ and transcriptional signatures related to IFN-γ production were linked to protection from infection.^92,93^ It is also conceivable that NK and NKT cells participate in IFN-γ-independent killing of infected hepatocytes. Recently, serological profiling studies suggested that NK cells may inhibit sporozoite invasion through antibody-mediated interactions.^94^ On the other hand, NKT cells may be dispensable in the development of sterile immunity.^95^

Nutritional immunity may play a role in protection against *Plasmodium* infections. In endemic settings, children with iron deficiency are protected against malaria.^96,97^ The hepatic hormone hepcidin has been reported to increase across the malaria season in these settings.^98,99^ Hepcidin restricts iron availability in the liver hence denying *Plasmodium* parasites a vital nutrient, and may protect against secondary liver-stage infections.^100^ Supplementing children with iron in a malaria-endemic region was associated with increased malaria incidences and mortality.^101^ Accordingly, targeting the nutritional requirements of the parasite is an alternative innate response to malaria infections.

### 3.2 Antibody responses, including targeting the parasites whilst in the skin

Antibodies are correlates of protection for most approved vaccines in clinical use. Their effector pathways include neutralization of pathogens, antibody-dependent cytotoxicity, antibody-dependent complement deposition and antibody-dependent phagocytosis. Mechanistically, humoral responses begin when a naïve B-cell encounters an antigen at the interface of the T and B regions of secondary lymphoid organs. Depending on the existing signals, these antigenically stimulated B cells may undergo (a) rapid proliferation in the extrafollicular foci to produce short-lived isotype-switched antibody-secreting plasmablasts (SLPCs), (b) interact with CD4^+^ T follicular helper (T_FH_) cells in a germinal-centre (GC)—dependent or GC-independent process to produce long-lived memory cells or (c) an anergic response. The B cells that interact with T_FH_-dependent differentiate into long-lived plasma cells (LLPC) or circulating memory B cells (MBCs) (as reviewed by Nutt et al^102^). LLPCs migrate to the bone marrow and continuously secrete neutralizing antibodies, whilst MBCs form a ready-to-respond antigen-specific B-cell pool.

Early malaria vaccine studies reported increased production of anti-CSP antibodies in response to RAS, and these antibodies are associated with protection against reinfection.^18,22,24,103^ In field and CHMI studies, antibody responses to other PE antigens such as LSA-1, TRAP and STARP have also been reported^104–106^ and protected individuals may have higher antibody titres.^105–107^ Passive transfer of monoclonal anti-sporozoite antibodies delayed patency of *Pb* infection in mice.^108^ The effector activity of these antibodies may include blocking sporozoite motility, dermal exit and subsequent invasion of hepatocytes.^78,109^ Antibodies may remove the surface coat protein of sporozoites in the skin and expose the parasites to their own pore-forming proteins.^110^ Beyond inhibiting sporozoite mobility, antibodies also aid in sporozoite destruction through activation of the complement system, phagocytosis and Fc-mediated innate cell functions.^94,111–113^

Various field studies have reported that high antibody levels against sporozoites are required for effective and long-term protection.^105,114,115^ RTS,S vaccines induce high anti-*Pf*CSP antibodies titres with moderate CD4^+^ T-cell responses,^116–118^ yet none of them have been recognized as an unequivocal correlate of protection. It remains poorly understood if protection against sporozoites is dependent on immunoglobulin sub-class, but high levels of antigen-specific IgG3 and IgG1 in participants receiving RTS,S have been observed.^111,119^ Although individuals with higher antibodies against sporozoite antigens have better protection against infection,^105–107^ antibody titres have generally performed poorly as correlates of protection in malaria vaccine studies.^94,120^ The modest efficacy of RTS,S in endemic regions suggests that the functionality and avidity of the antibodies, rather than the antibody titres, is a better correlate of immune protection to malaria.^94,113^ In recent serological profiling studies, the functionality of antibodies was reported to be a better predictor of protection.^94^ These antibodies were reported to induce NK cell effector functions, including activation and phagocytosis.

The hurdle with malaria infections is the inability to generate long-lasting protective immunity. This is compounded by the lack of appropriate surrogates of protection in field and CHMI studies. Malaria-specific MBCs are elicited at levels comparable to conventional licensed vaccines^121^ and can persist in naturally infected and travellers to endemic regions.^122^ Like antibodies, malaria MBCs appear to increase with age and exposure.^123^ Studies have demonstrated that *Pf*-specific MBCs target PE stage antigens, and existing antibodies to CSP, LSA-1 and TRAP may protect against clinical malaria in an endemic setting.^105,124^ Current literature does not indicate the magnitude of humoral reaction to other malaria PE antigens or if PE-specific MBCs are linked to protective immunity.

How antibody and MBC responses are regulated during malaria infections is poorly defined. T_H_1 responses have also been implicated in the regulation and function of MBCs after malaria infections in humans and mice.^125–127^ These studies reported that T_H_1-polarized PD-1^+^CXCR5^+^CXCR3^+^ T_FH_ cells are preferentially elevated during malaria infections and may play a role in impaired GC responses. How these responses influence LLPC and MBC responses to PE stages remain poorly characterized. Recently, a group of atypical MBCs (CD19^+^CD21^-^CD27^-^) expressing high levels of FcRL5 has been suggested to play a role in the incomplete anti-*Plasmodium* immunity.^128,129^ Whether or not atypical MBCs are induced during PE stage natural and vaccine responses remains to be described. However, the dynamics behind the MBC development and the roles of atypical MBCs in de novo malaria infections remain an open question.

### 3.3 CD4^+^ T-cell effector mechanisms

CD4^+^ T cells have multiple effector functions ranging from regulation of immune responses and activation of CD 8^+^ T cells, B cells, innate immune cells and other nonimmune cells.^130^ CD4^+^ T cells play a critical role in response to malaria PE stages and maintenance of immunity both independently and in conjunction with other cells.^131–133^ In model studies, CD4^+^ T cells were reported to confer protection against *Pb* and *Py* in β_2_-microglobulin knockout mice (CD8^+^ T cells deficient) immunized with RAS,^131^ probably through direct killing of infected hepatocytes.^134^ Field and CHMI studies have also reported high CD4^+^ T-cell numbers after RTS,S or whole sporozoite infection,^35,116,135^ including high serum levels of CD4^+^ T cell–associated cytokines (IFN-γ, tumour necrosis factor [TNF] and IL-2).^32,136^ In modelling and CPS vaccine studies, T cells^133^ and IFN-γ^92,93^ have been reported as correlates of immune protection against malaria infection. Detailed investigations are required to determine the longevity of CD4^+^ T cells in response to PE stages and their ability to serve as surrogates of immune protection.

The functional roles of CD4^+^ T cells are not limited to direct activity. As discussed before, CD4^+^ T cells may be involved in the licensing of the antigen-presenting DCs that prime effector CD8^+^ T cells. The cytokines generally produced by CD4^+^ T cells may influence other immune cells involved in response to malaria and development of immunity. IL-4–producing CD4^+^ T cells sustain and expand the effector and memory *Py*-specific CD8^+^ T-cell pool.^87,137,138^ In the absence of CD4^+^ T cells, the sporozoite-specific memory CD8^+^ T cells fail to protect against challenge infections in mice.^137^ Some of the cytokines produced by CD4^+^ T cells, such as IFN-γ, IL-4, IL-5 and IL-10, enable B cells to undergo immunoglobulin class-switching.^102^ A subset of CD4^+^ T cells, FOXP3^+^ regulatory T cells (T_REG_s), has been associated with poor development of CPS vaccine-induced immunity.^139^ A recent study implicated a subset of T_FH_ CD4^+^ T cells in the poor response of participants receiving RTS,S and ME-TRAP combinations.^77^ Nonetheless, further studies are required to elucidate induction, regulation, maintenance and tissue requirements of CD4^+^ T cells in malaria PE stage immunity.

### 3.4 CD8^+^ T-cell effector mechanisms, including liver-resident memory CD8^+^ T cells

CD8^+^ T cells are the primary effector cells against PE stages as seen in rodent, non-human primate and human studies.^140–144^ As observed in *Py*, the responses by CD8^+^ T cells begin after they are primed by mature CD11c^+^ DCs in the skin-draining lymph nodes.^8^ Naïve CD8^+^ T cells do not exert antiparasitic activity, unless previously primed by antigen-presenting cells.^145^ The CD8^+^ T cells with cognate receptors to the antigens presented by the DCs will differentiate to short-lived effector cells (SLEC) or memory precursor effector cells (MPEC) depending on the local cytokine environment and transcriptional factor profile.^146–148^ Activated CD8^+^ T then undergo clonal expansion, which requires the presence of IL-2/IL-4 produced by CD4^+^ T cells.^87^ The numbers of CD8^+^ T cells have been shown to increase rapidly after sporozoite inoculation.^86,145,149,150^ The activation and proliferation of naïve CD8^+^ T cells are dose-dependent, and a successful response requires viable sporozoites.^5,53,151^ The SLEC migrate to the liver to exert their effector properties whilst MPEC further differentiate to memory cells.^152,153^

CD8^+^ T cells confer sterile immunity against *Pb*-independent of B cells or CD4^+^ T cells.^18^ In rodent and nonhuman primate models, depletion of CD8^+^ T cells abrogates sterile immunity after RAS immunization, whilst their restoration reinstates the protection.^140,143^ However, the effector mechanisms of these malaria PE-specific CD8^+^ T cells are not well characterized. In vivo imaging studies report that CD8^+^ T cells recognize cognate epitopes on the infected hepatocyte MHC-I and cluster around these cells.^154^ Murine and vaccine studies have reported elevated CD8^+^ T-cell effector mediators including cytokines (IFN-γ and TNF) and/or proteins involved in contact-mediated cytotoxicity (perforin, TRAIL, FAS ligand and granzyme).^18,35,134,151,155^ Surprisingly, CD8^+^ T cells lacking perforin, FAS ligand and perforin can still eliminate *Py-* and *Pb-*infected hepatocytes.^156,157^

Malaria memory T cells are involved in patrolling, surveillance and rapid recruitment to the site of infection.^34,155,158^ This enables a fast, effective, specific and durable protection against subsequent malaria infections. Pre-clinical and CHMI trials have shown induction and persistence of *Pf*-specific CD4^+159,160^ and CD8^+^ T cells.^144^ In *Pb* and *Py*, CD8^+^ T memory cells have been described as CXCR3^hi^CXCR6^hi^ CD62L^–^CD69^+^ liver-resident (T_RM_), CXCR3^lo^CXCR6^lo^ CD44^+^CD62L^–^ CD122^–^ circulating effector (T_EM_), and CD44^+^CD62L^+^CD122^+^ central memory (T_CM_) cells,^157,161,162^ and their effector immune responses are species-specific.^157^ Nonetheless, the epitope signatures and correlates of CD8^+^ T memory cell protection are yet to be characterized.

Majority of the circulating CD8^+^ T memory cells in mouse studies are T_EM_ but a small proportion of T_CM_ has also been observed.^150,162^ A large population of T_EM_ cells is required for effective and longterm protection.^150,163^ Whilst T_EM_ rapidly induce effector functions, T_CM_ has been shown to respond to malaria challenge with delay and short-lived IFN-γ responses.^145,162^ T_RM_, on the other hand, are the non-circulating phenotype. T_RM_ cells have reduced expression of sphingosine 1 phosphate (S1P) receptor and CCR7, and have been associated with protection to sporozoite reinfection.^161^ In vitro studies suggest that the patrolling and effector activity of *Plasmodium*specific T_RM_ is dependent upon LFA1-ICAM1 interactions.^164^ Consequently, T_RM_ cells important in first-line responses including being able to recruit other cells despite the reduced ability to recirculate. Current efforts are underway to harness these T_RM_ for improved vaccines against PE stages.

### 3.5 Perspectives on immune responses to PE stages

Naturally acquired immunity in endemic areas is short-lived and non-sterilizing, and wanes over time without repetitive exposures. This suggests a defect in the development of immunological memory after natural malaria infections. The exact reason for this impaired immune memory has not been adequately described. Indeed, the induction, maintenance and regulation of effector and memory responses have emerged as crucial stumbling blocks in malaria PE stage vaccine development.

It is widely appreciated that an effective and long-lasting malaria vaccine will need to induce robust antibody and T-cell responses. This may require further investigations on the specificities and correlates of immune protection induced by vaccine and CHMI trials, as well how to maintain large frequencies of effector and memory responses. Studies from animal models and humans reiterate the need for extremely high titres of functional antibodies and elevated frequencies of CD8^+^ T cells for sterile protective immunity.^105,114,115,150,163^ There is paucity of data on the quantity of CD4^+^ T cells required to induce sterile immunity. More work is also needed to understand how trained immunity of innate cells, which has recently been described,^165,166^ may contribute to immune protection in PE stages. Various adjuvants including alum, ASO1 and viral vectors have been employed as immunostimulants and/or delivery systems for the existing vaccine candidates.^167^ Adjuvants have the potential to induce and maintain large numbers of effector and memory immune cells, and the appropriate choice or combination of adjuvants may be the key to unlocking a malaria vaccine that confers sterile and long-lasting protection.

Very little is known regarding the regulation of immune responses to PE stages—the possible roles for regulatory T cells, cytokines and T_H_1/T_FH_ have been thoroughly explored in malaria blood stages.^168^ Additionally, malaria blood-stage infections have been reported to downregulate PE stage immunity.^169,170^ Checkpoint blockade has been explored in cancer and malaria blood-stage research,^171^ and it is possible that some answers to the regulation of frequencies of anti-PE stage immune responses lie here. The contribution of inhibitory and other regulatory proteins, and their tissue-specific regulation, has not been widely studied in the context of malaria PE stages, but it is plausible that they are involved in a complex web of factors influencing protection against malaria.

## 4 Conclusion

Delivery of an efficient and long-lasting vaccine protection remains an ambitious goal that requires sustained efforts of all stakeholders. Gaps in the existing parasite-host immunological crosstalk in both the skin and the liver during malaria PE stages need to be addressed first. Quantification and characterization of immune mechanisms have only started to emerge recently despite decades of research into an efficient malaria vaccine. Nonetheless, the identification of correlates of protection and protective malaria PE stage epitopes remain a work in progress. In the current review, we highlighted how protection to malaria sporozoites may rely on a fine, yet to be adequately described, balance between innate and adaptive immune responses. Utilizing advances in other fields such as systems biology and bioinformatics can inform the study of more immunological processes, which have proven challenging to study in the setting of a natural infection. Alternative efforts should include targeting novel sporozoite proteins, a multi-stage and multi-antigen vaccine, or a ‘nutritional’ vaccine that targets metabolic requirements of sporozoites.

## Figures and Tables

**Table 1:** The status of current malaria pre-erythrocytic stage vaccine candidates (adapted from the World Health Organization tables of malaria vaccine projects globally—’Rainbow Tables’)^172^

Project	Registration no.	Sponsor	Vaccine type	Country	Phase	Start Date	Ref
Whole Sporozoite							
PfSPZ	NCT02215707	Sanaria Inc	RAS	USA	I	2014	51
PfSPZ	NCT02627456	Sanaria Inc	RAS	Mali	II	2016	
PfSPZ	NCT02613520	Sanaria Inc	RAS	Tanzania	I	2015	27,173
PfRAS	NCT01994525	USAMRDC	RAS	USA	I	2013	
PfSPZ-CVac	NCT02115516	Sanaria Inc	CPS (SPZ-CQ)	Germany	I	2014	54
PfGAP3KO	NCT03168854	NIAID	GAP	USA	I	2017	
PfSPZ	NCT02663700	NIAID	RAS	Burkina Faso, USA	I	2016	
PfSPZ-CVac	NCT02773979	NIAID	CPS (SPZ-CQ)	USA	I	2016	
Sub-unit							
RTS,S/AS01E	NCT02374450	GSK	CSP	Kenya, Burkina Faso, Ghana	IV	2015	174
RTS,S/AS01 fractional dose	NCT01857869	GSK	CSP	Kenya, Gambia, Burkina Faso	II	2013	61
R21/AS01B	NCT02600975	University of Oxford	CSP	United Kingdom	I	2015	
R21/Matrix – M1	NCT02925403	University of Oxford	CSP	Burkina Faso	I	2016	
R21/ME-TRAP	NCT02905019	University of Oxford	CSP/TRAP	United Kingdom	II	2016	175
CS-Vac	NCT01450280	University of Oxford	CSP	Ireland	I	2011	65
PfCelTOS FMP012/AS01B	NCT02174978	USAMRMC	CelTOS	USA	I	2014	
ChAd63/MVA ME-TRAP	NCT01635647	University of Oxford	ME-TRAP	Burkina Faso, Kenya, Gambia	II	2012	72-74
ChAd63/MVA ME-TRAP + Matrix M™	NCT01663512	University of Oxford	ME-TRAP	United Kingdom	I	2012	176
Adjuv R21 (RTS,S biosimilar) with TRAP combined	NCT02905019	University Oxford	ME-TRAP + CSP	United Kingdom, Germany	II	2016	

*Note*: RAS denotes radiation-attenuated sporozoites.

Abbreviations: Adjuv, adjuvant; CelTOS, cell-traversal protein for ookinetes and sporozoites; ChAd, chimpanzee adenovirus; CPS, chemoprophylaxis following sporozoite infection; CQ, chloroquine; CSP, circumsporozoite protein; GAP, genetically attenuated parasites; GSK, GlaxoSmithKline; KO, knockout; MVA, modified vaccinia Ankara; NIAID, National Institute of Allergy and Infectious Diseases; *Pf, Plasmodium falciparum;* SPZ, sporozoites; TRAP, thrombospondin-related anonymous protein; USAMRDC, United States Army Medical Research and Development Command.

## Data Availability

Data sharing is not applicable to this article as no data sets were generated or analysed during the current study.
